# Overexpression of carbonic anhydrase and HIF-1α in Wilms tumours

**DOI:** 10.1186/1471-2407-11-390

**Published:** 2011-09-12

**Authors:** Josiah V Dungwa, Linda P Hunt, Pramila Ramani

**Affiliations:** 1 Department of Histopathology, Bristol Royal Infirmary, Bristol BS2 8HW, UK; 2 School of Cellular and Molecular Medicine, University of Bristol, School of Medical Sciences, University Walk, Bristol BS8 1TD, UK; 3 Senior Lecturer in Medical Statistics, University of Bristol School of Clinical Sciences, UBHT Education Centre, Upper Maudlin Street, Bristol, BS2 8AE, UK

## Abstract

**Background:**

Overexpression of carbonic anhydrase (CA IX) is associated with poor survival in several adult-type cancers but its expression is undocumented in Wilms tumour (WT), the most common tumour of the paediatric kidney.

**Methods:**

*CA9 *expression was measured using polymerase chain reaction (PCR) in 13 WTs and matched-paired non-neoplastic kidneys (NKs). CA IX and hypoxia-inducible factor-1 α-subunit (HIF-1α) protein were quantified in 15 matched-paired WTs and NKs using enzyme-linked immunosorbent assays. CA IX and HIF-1α were localised by immunostaining tissue sections of 70 WTs (untreated WTs, n = 22; chemotherapy-treated WTs, n = 40; relapsed/metastatic WTs, n = 8). CA IX-positive untreated WTs (n = 14) were immunostained for vascular endothelial growth factor (VEGF), glucose transporter-1 (GLUT1) and CD31. Double staining for CA IX and CD31 was performed in WTs (n = 14).

**Results:**

*CA9 *full length (FL) was significantly up-regulated in WTs compared to NKs (*p *= 0.009) by real-time PCR. Conventional PCR showed expression of alternative splice variant in all NKs and WTs but FL in WTs only. WTs showed a 2-fold increase in CA IX protein over NKs (*p *= 0.01). HIF-1α levels were up-regulated in WTs compared to NKs, although the difference was not statistically significant (*p *= 0.09). CA IX and HIF-1α immunolocalisation were observed in 63% and 93% of WTs, respectively. The median fraction of cells staining positively for CA IX and HIF-1α was 5% and 22%, respectively. There was no significant association between the expression of either CA IX or HIF-1α and clinicopathological variables in WTs resected following chemotherapy. VEGF and GLUT1 immunoreactivity was seen in 94% and 100% with the median fraction of 10% and 60% respectively. Co-expression and co-localisation of all four hypoxia markers was seen in 7/14 and 6/14 cases respectively. CA IX was seen in well vascularised areas as well as in the peri-necrotic areas.

**Conclusions:**

Carbonic anhydrase 9 (mRNA and protein), and HIF-1α protein are overexpressed in a significant portion of WTs. No significant association was detected between the expression of either CA IX or HIF-1α and clinicopathological variables in WTs resected following chemotherapy. Cellular localisation studies in untreated WTs suggest that CA IX and HIF-1α are regulated by hypoxia and non-hypoxia mechanisms.

## Background

Wilms tumour (WT) is the most common paediatric renal tumour, with an incidence of 1:10,000 children. Although the prognosis of children with favourable clinical and pathological factors is good, children with high-risk, drug-resistant or metastatic disease have a mortality rate of 10-15%. Alternative therapeutic strategies are therefore essential to improve the outcomes of the children in this group.

Tissue hypoxia contributes to the progression and chemoresistance of many human cancers by inducing the up-regulation of genes associated with angiogenesis, metabolism, cell survival and apoptosis [1]. The cellular response to hypoxia is mediated by a key gene, hypoxia-inducible transcription factor-1 α-subunit (HIF-1α), via hypoxia response elements in the promoter regions of target genes, which include carbonic anhydrase IX (*CA9*) and vascular endothelial growth factor (*VEGF*)[1]. CA IX, a membrane glycoprotein, plays an important role in the growth and survival of tumour cells under both normoxic and hypoxic conditions [2,3]. Although hypoxia plays a major role in HIF-1α stabilisation and activation, the amplitude of the response can be modulated in normoxia by growth factors, oncogenes and cytokines [4].

Up-regulation of HIF-1α and CA IX is observed in a variety of epithelial cancers and correlates with poor survival [1,5]. Epithelial cancers typically occur in adults and only rarely occur in children. In contrast, solid tumours (such as WTs) that occur in children are developmental disorders presenting different biological characteristics [6]. In a study consisting of 18 WT samples, HIF-1α and VEGF expression was detected in all WTs using immunohistochemistry [7]. In a recent study, up-regulation of *CA9 *in WTs was associated with high-risk histology and was significantly correlated with metastases [8]. The existence of two variants of *CA9*, full length (FL) and alternate spliced variant (ASV), has been described in both hypoxic and non-hypoxic conditions [9,10]. The ASV mRNA, which lacks the exons 8-9, competes with FL in regulating extracellular pH in mildly hypoxic conditions. ASV is expressed in both normal and diseased tissues, while FL mRNA is upregulated in non-small cell lung cancer and its expression associated with poor prognosis [10]. Wittmann et al used primers that did not distinguish between the two isoforms of *CA9 *[8].

As CA IX is an attractive target for anti-cancer therapy for several reasons, overexpression of *CA9 *[8] is an important finding that is likely to be clinically relevant [11,12]. Although changes in protein expression are more likely to have a direct impact on biological behaviour than changes in transcriptional regulation or mRNA expression, there is no information about CA IX expression in WTs. The aims of this study were as follows: 1) to perform a comprehensive expression analysis of carbonic anhydrase at mRNA (FL and ASV *CA9*) and protein levels and HIF-1α protein in matched-paired WTs and non-neoplastic kidneys (NKs), precursor lesions (nephrogenic rests) [13], and fetal kidneys; (2) to look for associations between the expression of either CA IX or HIF-1α and clinicopathological features; and (3) to compare the expression of CA IX with other hypoxia markers and vascularity.

## Methods

### Tissue samples

The study was approved by the South Bristol and North Somerset Research Ethics Committee (09/H0106/4). The clinicopathological information, including the size, weight, histological type, stage and risk group, of 70 WTs was extracted from pathology reports. All samples were obtained from the Paediatric Pathology files of the University Hospitals Bristol NHS Foundation Trust.

The tissue specimens were taken from 62 primary and 8 metastatic/relapsed WTs. The primary WTs were obtained from patients undergoing nephrectomies without chemotherapy (n = 22) and nephrectomies that were performed after standard chemotherapy (*n *= 40) according to the appropriate United Kingdom Wilms tumour and Société Internationale d'Oncologie Pédiatrique protocols (reviewed by [14]). The metastatic sites included lymph nodes (*n *= 2), the liver (*n *= 2) and lungs (*n *= 4). Three WTs demonstrated focal anaplasia (intermediate risk) and three demonstrated diffuse anaplasia (high-risk).

Areas of necrosis in 19/22 untreated WTs were included for immunohistochemical (IHC) staining because markers of perinecrotic localisation have been studied in several cancers to elucidate their regulation under hypoxic conditions in animals [15] and humans [16-19]. 40 blocks from post-chemotherapy nephrectomy specimens were selected as anti-CA IX and/or HIF-1α therapies [20] are likely to be used in this clinical setting. Selected blocks contained representative areas of the WT and adjacent non-neoplastic kidney for comparison. A total of 26 nephrogenic rests were included in the study. Sections from 5 fetal kidneys ranging from 12- to 30-weeks' gestation were also examined by IHC. Frozen tissue with at least 70% viable tumour was available from 15 post-chemotherapy WTs. Frozen tissue from 15 matched-paired NKs were also available.

### RNA extraction

Total RNA was extracted from 26 samples (13 WTs and 13 matched-paired NKs). RNA was extracted using TRIzol according to the manufacturer's protocol (Invitrogen, Paisley, Scotland, UK) and quantified on a NanoDrop ND-1000 Spectrophotometer (Thermo Fisher Scientific, Loughborough, UK).

### Reverse transcription polymerase chain reaction

10 μg of RNA was reverse transcribed using the qScript reverse transcription kit (Primerdesign, Southampton, UK) as per manufacturer's protocol. The presence of *CA9 *isoforms in our samples was evaluated using two sets of primers as previously described by Barathova et al [9]. The FL isoform was identified using the h7S-h8A primer set while the h7S-h10/7A set was used to detect the ASV. cDNA was amplified for all PCR reactions in a final volume of 25 μl containing PCR Master mix 5× (Promega, Southampton, UK), 1 μM each of forward and reverse primers at the following conditions: 10 min at 95°C, 30 s at 95°C, 30 s at 62°C and 30 s at 72°C for 35 cycles and a final extension at 72°C for 7 min for FL primers. Annealing for the ASV primers was performed at 54°C for 30 s. Normal stomach which contains both isoforms was used as a positive control. All products were resolved by electrophoresis on a 2.5% ethidium bromide stained Agarose Micro Plus gel (alpha laboratories, Eastleigh, UK). After visualisation, amplicons were extracted from the gel according to the instructions of the Qiagen Gel Extraction kit (Qiagen, Crawley, UK). 20% of the products were then sequenced to confirm their identity and localise the splicing site.

### Real-time quantitative polymerase chain reaction

RNA quality analysis was performed using the Bio-Rad Experion System (Bio-Rad Labs, Hemel Hempstead, UK). Universal mRNA (AMS Biotechnology, Abingdon, UK) served as the positive control. No reverse transcriptase and no template served as the negative controls. A total of 1 μg of RNA was used for each RT reaction in a final volume of 20 μl using the iScript cDNA Synthesis kit and cycling conditions specified by the manufacturer (Bio-Rad Labs, Hemel Hempstead, UK). Real-time quantitative polymerase chain reaction (q-PCR) for *CA9 *was performed using a Roche LightCycler 480 (Roche Diagnostics Gmbh, Mannheim, Germany). The cDNA was analysed by q-PCR using the LC480 probe mastermix (Roche Diagnostics Limited, West Sussex, UK) and detected on the FAM channel. The sequences of the sense and antisense PerfectProbe (Primerdesign, Southampton, UK) for FL (accession number: NM_001216) were 5'-GCTCCACACCCTCTCTGAC-3' and 5'-CTCAATCACTCGCCCATTCAA-3', respectively. The size of the amplicons was 97 bp. The amplification conditions consisted of an initial denaturation step of 95°C for 10 min, followed by 50 cycles of denaturation at 95°C for 15 s, annealing at 50°C for 30 s and extension at 72°C for 15 s.

Relative expression of the transcripts was quantified after normalising the levels with the geometric mean of three endogenous reference genes, *PPIA, GUS *and *TBP*. These genes were found to have the least variation among 12 reference genes (the TATAA human endogenous control gene panel, Göteborg, Sweden) in a preliminary experiment. Data analysis was performed using GenEx 5.0 (MultiD, Göteborg, Sweden), which uses the ΔΔCt relative quantification model with PCR efficiency correction and reference gene normalisation.

### Protein extraction

Total protein was extracted from 15 WTs and 15 matched-paired NKs. Snap-frozen tissue samples were homogenised in ice-cold lysis buffer composed of 1% Triton X-100 buffer containing 1:100 dilutions of protease and phosphatase inhibitor cocktails (Sigma-Aldrich, Dorset, UK). After homogenisation, the samples were centrifuged at 12,000 × *g *for 15 min at 4°C. Total protein was quantified using the Bradford assay following the manufacturer's instructions (Sigma-Aldrich, Dorset, UK).

### Western blotting

A total of 40 μg/sample of protein was denatured by boiling at 100°C in Laemmli buffer for 5 min, separated using a 12% SDS-PAGE gel (NuPage Novex Bis-Tris mini gels, Invitrogen, Paisley, Scotland, UK) and transferred onto polyvinylidene fluoride (PVDF) membrane (Immobilon membranes, Millipore, Watford, UK). CA IX and HIF-1α were detected by immunoblotting using mouse anti-CA IX (1:50, M75) and mouse anti-HIF-1α (1:500, clone ESEE122, Abcam, Cambridge, UK) for 2 h. After washing with PBS-Tween (0.05%), the membranes were incubated with horseradish peroxidase-conjugated goat anti-mouse IgG at 1:5000 (Thermo Fisher Scientific, Loughborough, UK) for 2 h at RT. Immunodetection of the protein was performed using an enhanced chemiluminescence (ECL) kit following the manufacturer's instructions (Immobilon Western Chemiluminescent HRP Substrate, Millipore, Watford, UK). Immunoreactive bands were visualised on Hyperfilm ECL (GE Healthcare, Chalfont St. Giles, Bucks, UK), and the predicted protein was confirmed by recombinant CA IX and HIF-1α that was run alongside the samples. To control for protein loading, the membranes were stripped and re-probed with anti-GAPDH (1:5000, AF5718, R&D Systems, Abingdon, UK).

### Enzyme-linked immunosorbent assay (ELISA)

CA IX and HIF-1α concentrations were measured in WT and matched-paired NK samples using the human CA IX Duoset and HIF-1α Duoset IC enzyme-linked immunosorbent assay kits (DY2188 and DYC1935-5, respectively, R&D Systems, Abingdon, UK), according to the manufacturer's instructions. Standard curves were constructed using 2-fold serial dilutions of recombinant CA IX (range 0 - 2000 pg/ml) and HIF-1α (range 0 - 8000 pg/ml). Optical densities were determined using a spectrophotometer at λ = 450 nm with corrective reading at λ = 570 nm. Each protein extract was measured three times, and the tests were performed in triplicate each time. Both the intra-assay and inter-assay variation were < 10%.

### Immunohistochemical techniques

4 μm thick conventional (whole) sections of formalin-fixed paraffin-embedded tissues were stained using a BOND-III automated immunostainer (Leica Biosystems, Milton Keynes, UK). Anti-CA IX (1:50), anti-HIF-1α (1:2500), mouse anti-VEGF (1:300, VG1, Abcam, Cambridge, UK), and rabbit anti-GLUT1 (1:800, AB1340, Merck Millipore, Watford, UK) were applied to the sections for 1.5 h. The Bond™ Polymer Refine Detection system (DS9800, Leica Novocastra, Milton Keynes, UK) was used. Normal appendix tissue was used as a positive control for CA IX, breast carcinoma tissue served as a control for HIF-1α, VEGF and GLUT1. Using the same concentration as the primary antibodies, non-immune isotype IgG was used as a negative control.

To study the precise relationship between CA IX and vascularity, double immunostaining was performed on 14 pre-chemotherapy WT samples that showed necrosis and for which unstained sections were available. To achieve this, CA IX and mouse anti-CD31 (1:100, M0823, Leica Novocastra, Milton Keynes, UK) antibodies were incubated for 2 h and 4 h followed by detection using the Bond™ Polymer Refine Detection system and Bond™ Polymer Refine Red Detection system (DS9800 and DS9390, Leica Novocastra, Milton Keynes, UK) respectively.

The percentage of cells expressing CA IX, HIF-1α, VEGF and GLUT1 was recorded in each of the three WT components: blastema, epithelium and stroma. Weak or focal staining in < 1% of the sections was considered negative. Positive nuclear or cytoplasmic HIF-1α staining was graded by the intensity (weak, moderate or strong), all of which were considered positive. Because CA IX, VEGF and GLUT1 staining was of uniformly strong intensity in all three WT components, the percentage of cells stained for each element was recorded, but the intensity was not scored.

### Statistical analyses

The means of the duplicate mRNA expression profile data were used for analysis. Relative expression levels from the PCR were positively skewed; therefore, they were logarithmically transformed prior to statistical analysis and geometric means (CIs) used for data summary. The (log-transformed) expression values of paired NK and WT samples were compared using paired Student's t-tests.

For the immunohistochemical scores, chi-squared tests were used to compare groups exhibiting any positive staining. Yates' continuity correction was used for 2 × 2 tables. Fisher's Exact tests (two-tailed) were used where expected frequencies were small.

For the ELISA comparisons, averages of triplicate measurements were used, again after logarithmic transformation to remove positive skewness. Paired Student's t-tests were used to compare the expression in 1:1 paired normal and WT samples. Mixed effects regression models were used to explore the relationship between CA IX and HIF-1α. A 5% level of significance was used throughout the analyses.

## Results

### *CA9 *isoforms are expressed in WT and NK

**Figure 1:**
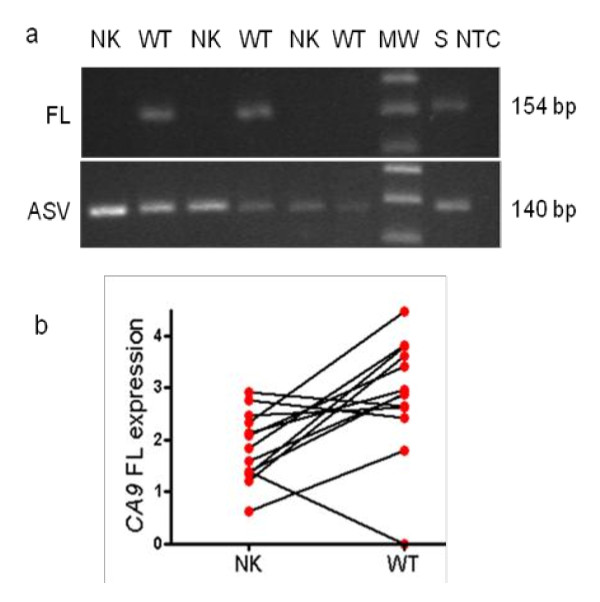
**a) RT-PCR analysis of *CA9 *isoforms in paired non-tumoral kidneys (NK) and Wilms tumours (WTs)**. Upper panel: *CA9 *full length (FL), (154 bp using primers h7S-8A), Lower panel: *CA9 *alternative splice variant (ASV) (140 bp using primers h7S-h10/7A). Stomach (S) was used as a positive control for both FL and ASV CA9. No test control (NTC). b) The mean relative expression of *CA9 *FL in 13 matched-paired NK and WTs. Total RNA was extracted from NK and WT samples. Each data point indicates log-transformed relative mRNA expression levels normalised to the lowest expression. *CA9 *FL expression was significantly higher in WTs than in NKs (*p *= 0.009). MW = molecular weight marker.

On qualitative PCR analyses, CA9 FL was detected only in WTs while the ASV isoform was detected in all the samples (Figure 1a upper panel and lower panel, respectively). On sequencing the recovered PCR products, the expected sequences were obtained.

### *CA9 *expression is significantly up-regulated in WTs

Using quantitative PCR, there was significant up-regulation of *CA9 *FL in 13 matched-paired WTs and NKs (Figure 1b, p = 0.009 using log-transformed values). The mean of the relative expression in the paired samples was 2.87 [median, 2.89; SE, 0.31] in the WTs and 1.85 [median, 1.84; SE, 0.19] in the NK samples, indicating a 1.55-fold higher expression in the former.

### CA IX and HIF-1α are expressed in WTs

**Figure 2:**
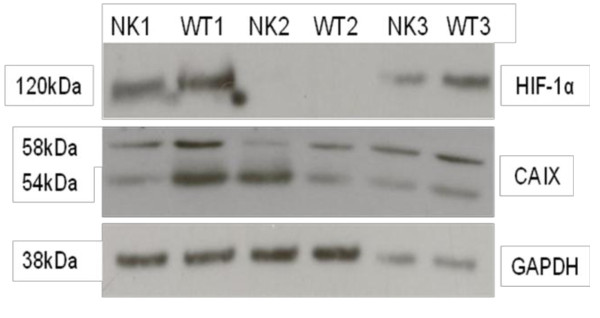
**Protein expression in cell lysates from matched-paired NK and WT samples as detected by western blot analysis**. Figure 2a. HIF-1α (upper panel) and CA IX (middle panel) and the lower panel indicates even protein loading as evidenced by detection of the GAPDH control from the same extract. GAPDH is visible as a band of approximately 38 kDa. Upper panel: A single band at 120 kDa indicates full length HIF-1α protein detected in two post-CT samples (pairs 1 and 3). HIF-1α is higher in WTs than in matched-paired NKs. Middle panel: CA IX expression, indicated by two bands of 54 kDa and 58 kDa, is higher in two out of three WTs compared to their corresponding NKs.

In preliminary experiments, we performed western blotting to confirm the expression and specificity of the antibodies used in our laboratory (Figure 2). HIF-1α (Figure 2 upper panel) was expressed in WTs and NKs. Translation of *CA9 *into CA IX was evidenced by the characteristic twin bands at 54 and 58 kDa (Figure 2 middle panel).

### CA IX and HIF-1α immunolocalisation in WTs, nephrogenic rests (NRs), fetal kidneys and non-neoplastic kidneys (NKs)

Immunolocalisation studies were performed in all 70 WTs to examine the distribution of hypoxia markers. The number of WTs stained with CA IX and HIF-1α, including the proportions of the individual cellular components, is presented in Table 1.

**Table 1:** Immunolocalisation of CA IX and HIF1-α in WTs (n = 70)

	%WT stained	Median % of all cells stained (range)	Blastema %	Epithelium %	Stroma %	Anaplastic cells %
CA IX	63	5 (0-40)	5	10	15	0
HIF1-α	93	22 (0-75)	24	28	1	0

**Figure 3:**
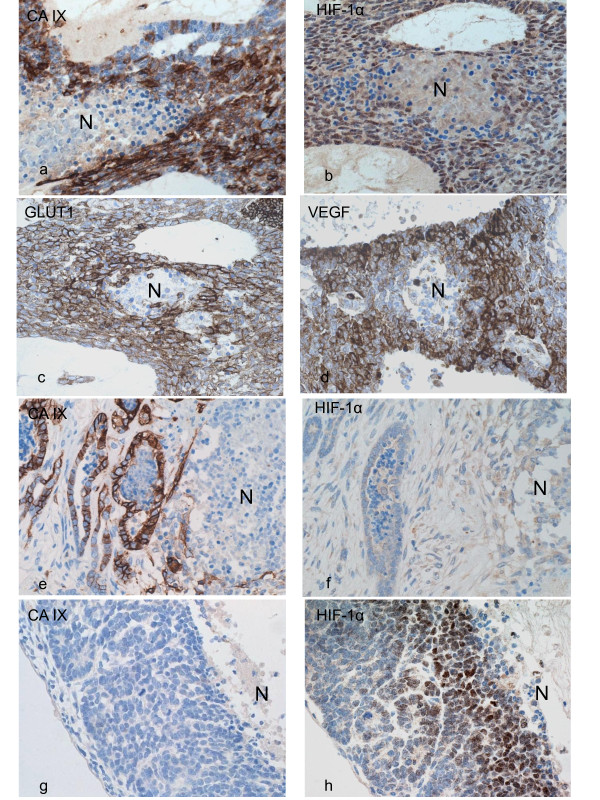
**Immunohistochemical staining for CA IX (a, e, g), HIF-1α (b, f, h), GLUT1 (c) and VEGF (d) in WTs**. Co-localisation of CA IX, HIF-1α, GLUT1 and VEGF in perinecrotic blastema (a-d). Perinecrotic tubules show CA IX but not HIF-1α expression (e, f). Perinecrotic blastemal cells are negative for CA IX, but nuclear expression of HIF-1α is present (g, h).

CA IX: 44/70 (63%) WTs expressed CA IX. Strong membranous/cytoplasmic immunoreactivity was detected in at least one of the three WT components (Figures 3a, 3e), whereas NRs, NKs and fetal kidneys were essentially negative.

HIF-1α: 65/70 (93%) WTs expressed HIF-1α. Two patterns of immunostaining were observed in the blastemal and epithelial components, nuclear and cytoplasmic. Nuclear expression of moderate to strong intensity was observed mainly in the perinecrotic areas (Figure 3b, h), whereas cytoplasmic staining was more diffusely distributed and generally demonstrated weak intensity (Figure 3f).

The staining of fetal kidneys revealed weak, diffuse immunoreactivity in the metanephric blastema, tubules and glomerular podocytes but no stromal expression was seen. All the NKs showed HIF-1α immunoreactivity located predominantly in the tubules and collecting ducts. Occasional glomerular podocyte staining was also observed. Tubular staining of variable intensity in the cytoplasm was observed in 20 of 26 nephrogenic rests, ranging from weak to moderate in intensity.

### Relationship of CA IX with hypoxia markers

Untreated WTs (n = 22) were immunostained to avoid bias due to direct toxic effects of drugs in WTs resected following chemotherapy. To study the relationship of CA IX, HIF-1α, GLUT1 and VEGF with hypoxia, 14 WTs containing areas of necrosis were assessed. The results are summarised in Table 2. Perinecrotic coexpression of all four markers was seen in 7/14 (50%) while colocalisation of all four was seen in 6/14 (43%) (Figures.3a-d).

**Table 2:** HIF-1α, CA IX, GLUT1 and VEGF expression in pre-chemotherapy WTs

Marker	Tumours +ve (%)	Level of expression (median)	Pattern of co-expression														
CA IX	56	0-60 (5)	+	+	+	+	-	-	-	-	+	+	-	-	+	-	+
HIF-1α	94	0-80 (40)	+	+	+	-	+	-	-	-	+	-	+	+	-	+	-
GLUT1	100	30-80 (60)	+	+	-	+	+	+	-	-	-	-	+	-	+	-	-
VEGF	94	5-90 (10)	+	-	+	+	+	+	+	-	-	-	-	-	-	+	+
Co-expression^a^			7	1	0	1	5	0	0	0	0	0	0	0	0	0	0
Co-localization^b^			6	1	0	0	4	0	0	0	0	0	1	0	0	0	0

Table shows expression in pre-chemotherapy WT samples that were CA IX positive (n = 14).

^a ^number of positive cases showing specific staining of all four markers (+ or - sign in each line indicates positivity and negativity in respective staining in the same tumour)

^b ^number of positive cases showing specific staining of all four markers (+ or - sign in each line indicates positivity and negativity in respective staining in the same area of the same tumour)

### Relationship of CA IX with vascularity

**Figure 4:**
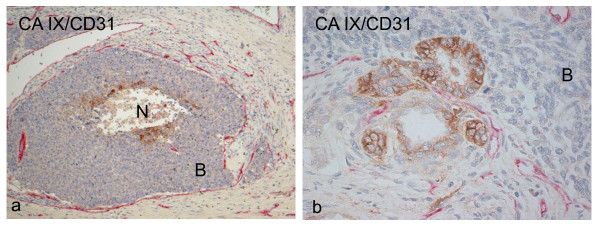
**Perinecrotic staining of CA IX (brown) away from CD31-stained vessels in red (a)**. Membranous CA IX staining (brown) of tubules adjacent to blood vessels highlighted by CD31 staining (red) (b). N = necrosis; B = blastema.

Of the 14 WT samples that were double stained, all showed perinecrotic CA IX upregulation away from CD31 stained vessels (Figure 4a). 3/14 (22%) showed CA IX expression adjacent to CD31 stained vessels (Figure 4b).

### CA IX and HIF-1α levels are higher in WTs than in matched NKs

**Figure 5:**
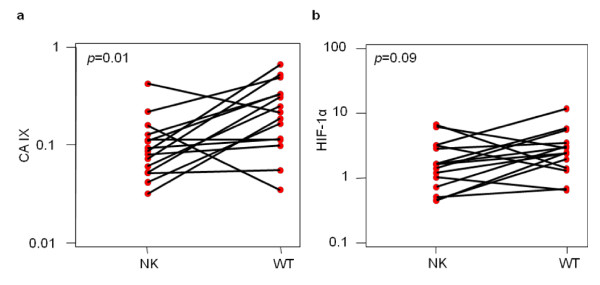
**CA IX and HIF-1α protein levels in total cell lysates of 15 Wilms tumours (WTs) and 15 matched-paired uninvolved kidney (NK) samples as quantified by ELISA**. a) CA IX is significantly higher in WTs. b) HIF-1α is up-regulated in WTs compared to paired NKs. Paired results are plotted on a log scale.

Because IHC is a subjective and semi-quantitative technique, CA IX and HIF-1α levels were quantified in the protein extracts of 15 WT and matched-paired NK samples using sensitive ELISAs (Figure 5a, b). The geometric mean of the CA IX levels was 0.09 pg/μg protein [95% CI: 0.06-0.13] in NKs and 0.20 pg/μg [95% CI: 0.13-0.31] in WTs. Compared to the matched-paired NKs, CA IX levels were significantly higher in WTs (*p *= 0.012, paired t-test using log-transformed values).

**Figure 6:**
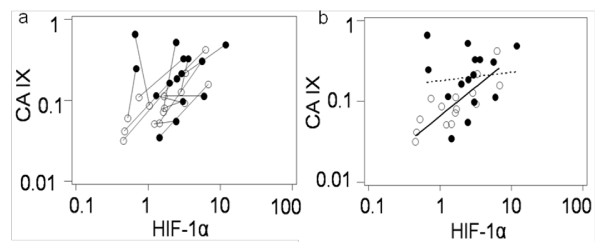
**Relationship between CA IX and HIF-1α protein levels in cell lysates of 15 Wilms tumours (WT) and 15 matched-paired uninvolved kidney (NK) samples as quantified by ELISA**. Open and closed circles indicate NK and WT samples, respectively. a) Pairs of results from the same patient are linked. b) Regression lines fitted to the NK (continuous line) and WT (dotted line) samples using a mixed regression model.

The geometric mean of the HIF-1α levels was 1.56 pg/μg total protein [95% CI: 0.96-2.52] in NKs and 2.49 pg/μg [95% CI: 1.62-3.81] in WTs. The difference, however, was not statistically significant (*p *= 0.093, paired t-test using log-transformed values). The trend indicating increased CA IX protein levels together with increased HIF-1α levels in WTs compared to NKs is illustrated in Figure 6a, which links the paired NK and WT samples.

### CA IX and HIF-1α correlation

After log transforming the NK sample data, CA IX expression demonstrated a strong positive correlation with HIF-1α [*r *= 0.82, 95%CI: 0.52-0.94, *p *< 0.001]. The estimated regression slope for CA IX on HIF-1α was 0.63 [95%CI: 0.36-0.89]. As depicted in Figure 6b, which provides separate regression lines for the NK and WT samples fitted using mixed model regression, the relationship among the WT samples was weaker; however, the slopes did not differ significantly (*p *= 0.052).

### Relationship between CA IX and HIF-1α cellular expression and clinicopathological variables in WTs

No significant difference in either CA IX or HIF-1α expression was detected between primary and relapse/metastatic WTs (Table 3). Similarly, there was no significant association between CA IX or HIF-1α cellular expression and the other variables, including histological type, stage, risk-group, size of WTs and kidney weight. Additionally, no significant difference was detected in either CA IX or HIF-1α cellular expression in pre-chemotherapy versus post-chemotherapy WT samples.

**Table 3:** Relationships between CAIX and HIF-1α & clinicopathological variables

*Characteristic *	No	No (%) stained for CAIX	p-value	No (%) stained for HIF-1α	p-value
*Relapse/metastases*					
Present	8	4	> 0.99	6	0.49
Absent	62	40 (65%)		59 (95%)	
*Chemotherapy status*					
Pre-CT	22	16 (73%)	0.47	20 (91%)	0.29
Post-CT	40	24 (60%)		39 (98%)	
*Stage *Post-CT					
1	14	9 (64%)	0.85	14 (100%)	†
2	13	7 (54%)		13 (100%)	
3/4/5	13	8 (62%)		12 (92%)	
*Risk group *Post-CT					
High-risk	5	2	0.37	5	> 0.99
Low/Intermediate risk	35	22 (63%)		34 (97%)	
*Histological type *Post-CT					
Blastemal	8	3	†	8	†
Epithelial/Stromal	10	9 (90%)		10 (100%)	
Mixed	16	8 (50%)		15 (94%)	
Regressive	6	4		6	
*Size (cm) Post-CT			> 0.99		0.45
≤9	22	13 (59%)		22 (100%)	
> 9	18	11 (61%)		17 (94%)	
**Weight (g) Post-CT					
≤299	20	7 (35%)	0.057	17 (85%)	> 0.99
> 299	20	14 (70%)		17 (85%)	

CT - chemotherapy, * size - 9 cm is the median size, **weight - 299 g is the median weight of total nephrectomy specimens. In 3 cases weight was unavailable, while 2 were partial nephrectomies.

†expected frequencies too small to do χ^2^-test

8 WT samples obtained from metastatic sites in addition to their primary tumours. Repeated samples from the same patient were regarded as independent samples in these comparisons.

## Discussion

This study is the first to assess the cellular distribution of CA IX protein and quantify it in Wilms tumours (WTs). The main strengths of our study include the use of a wide range of techniques for expression analysis and the comparison between WTs and matched-paired NKs to reduce the confounding variation inherent in biological samples. Our study clearly demonstrates that a) CA IX is over-expressed in up to 63% of WTs at the cellular level and b) CA9 FL and CA IX levels are significantly increased in WTs compared with NKs. We also demonstrate that HIF-1α protein is up-regulated in WTs compared to NKs. The present study also demonstrates the co-expression of CA IX with other hypoxia markers such as VEGF and GLUT1 in addition to HIF-1 α. The presence of CA IX in well vascularised areas, as shown by double staining of CD31 with CA IX suggests that CA IX is regulated by other factors other than hypoxia.

By using the M75 antibody, which recognises the extracellular proteoglycan-like domain of CA IX and is considered the gold standard in immunohistochemical CA IX detection, CA IX was detected in all three WT components. In our samples, the stained tumour fraction was 5%, an observation in agreement with the low frequencies previously described by others [19,21]. Generally, the method used to establish the prognostic significance of HIF-1α and CA IX expression in other tumours is immunohistochemistry [1,5]. Intratumoral heterogeneity, however, is a feature of CA IX cellular detection that is emphasised by this study and has been reported for other tumours [21,22]. In a seminal study of 110 previously treated cervical cancers, measurement of CA IX in multiple biopsies indicated that intratumoral heterogeneity accounted for 41% of the total variance in the data set [21]. We used conventional, whole tissue sections to reduce the inherent sampling error resulting from distributional heterogeneity of CA IX [22] and in small biopsies. We were unable to detect a significant association with clinicopathological variables, including metastatic status. A possible explanation for the lack of association is that there were too few samples in some subgroups to effectively evaluate the relationship between CA IX expression and these variables.

We showed *CA9 *ASV expression using qualitative PCR in all NKs and WTs in keeping with previously published data, [9,10]. We also showed an increase in *CA9 FL *in WTs compared to NKs using quantitative PCR. Our results are in line with increased CA9 FL expression shown in non-small cell lung cancer samples [10]. As CA9 FL was not expressed or expressed in very low amounts in normal kidneys, our study also indicates that it is a tumour-associated gene, which is an important finding because the other genes detected in WTs and nephrogenic rests are usually expressed during organogenesis [13]. The precise role of CA IX in WTs, however, remains to be elucidated. A recent study demonstrated that hypoxia down-regulates several genes involved in maintaining cell-cell interactions, including E-cadherin, which promotes epithelial to mesenchymal transition and invasion [23]. The proteoglycan-like region at the N-terminus of CA IX is implicated in cell adhesion [24,25], and CA IX's distribution overlaps with that of E-cadherin in canine kidney epithelial cells *in vitro *[26]. Furthermore, CA IX co-precipitates with β-catenin, reduces the binding of E-cadherin to β-catenin and thereby destabilises intercellular adhesion [26]. Importantly, E-cadherin expression is not only decreased in WT [27-29] but is also associated with advanced-stage disease [30]. Thus, it is possible that CA IX destabilises E-cadherin's linkage to the cytoskeleton, resulting in decreased cell-cell adhesion and contributing to WT invasion.

The cellular overlap between HIF-1α and CA IX in perinecrotic regions indicates that both are regulated by hypoxia in WTs. The localisation of CA IX in areas of tumour necrosis is of particular interest in terms of hypoxia regulation [15-19,31]. HIF-1α is the key transcriptional regulator of CA IX, and both co-localise in many embryonic and fetal tissues. A lack of co-expression in other tissues, however, indicates that CA IX is only partially regulated by HIF-1α expression. For example, HIF-1α, but not CA IX, is expressed in the human fetal and adult kidney as detected by immunohistochemistry [32,33]. Although a predominant perinecrotic pattern was observed in our study, CA IX was also present in areas distant from necrosis. This partial correlation is likely due to differences in the kinetics of HIF-1α and CA IX. HIF-1α is rapidly induced and degraded within minutes of re-oxygenation, whereas CA IX, induced hours later, is relatively stable and has a half-life of 2-3 days [34].

HIF-1α and CA IX expression in perivascular areas is in agreement with their regulation by hypoxia-independent-pathways in WT and in line with previous studies of epithelial cancers [15-19,31]. Mild hypoxia generated in high-density cultures can also increase HIF-1α synthesis and CA IX expression via the phosphatidylinositol 3-kinase (PI-3K) pathway [35]. Extracellular acidosis, a common feature in tumours, increases HIF-1α protein accumulation and *CA9 *transcriptional activity under normoxic conditions [36]. Additionally, growth factors, such as transforming growth factor-α (TGF-α), can also increase CA IX expression by increasing HIF-1α synthesis [35] via both the PI-3K and mitogen-activated protein kinase pathways [4]. In contrast to regulation under hypoxic conditions, which affects all cells by stabilising HIF-1α protein and increasing HIF-1 transactivation function, this regulation is cell-type specific and works mainly by increasing HIF-1α synthesis [4]. Although neither activating mutations [37] nor the amplification of EGFR [38] have been detected, increased TGF-α and EGFR expression is observed in WTs at both the mRNA [8] and histological levels [39].

## Conclusions

Our expression data clearly demonstrate that carbonic anhydrase (mRNA and protein) is up-regulated in untreated and treated WTs as compared with normal kidneys and WT precursor lesions (nephrogenic rests). We did not detect significant associations between either CA IX or HIF-1α protein and clinicopathological variables, including metastatic status in post-chemotherapy-treated WTs. Cellular localisation studies in untreated WTs suggest that CA IX and HIF-1α are regulated by hypoxia and non-hypoxia mechanisms.

## Abbreviations

CA: carbonic anhydrase; *CA9*: carbonic anhydrase 9 gene (including any genomic sequence and mRNA); CA IX: carbonic anhydrase 9 protein; HIF-1α: hypoxia-inducible factor; WT: Wilms tumour; NK: uninvolved/non-neoplastic kidney; NR: nephrogenic rest.

## Competing interests

The authors declare that they have no competing interests.

## Authors' contributions

PR conceived the study, secured the funding, selected the tumour samples, analysed the RNA expression data and wrote the paper. JVD conducted the assays. JVD and PR assessed the immunolocalisation and analysed the data. LPH performed the statistical analyses and contributed to the writing of the methodology and results sections of the paper. All the authors read and approved the final manuscript.

## Pre-publication history

The pre-publication history for this paper can be accessed here:

http://www.biomedcentral.com/1471-2407/11/390/prepub
